# Sport participation settings: where and ‘how’ do Australians play sport?

**DOI:** 10.1186/s12889-020-09453-3

**Published:** 2020-09-03

**Authors:** R. Eime, J. Harvey, M. Charity

**Affiliations:** 1 School of Science, Psychology and Sport, Federation University Australia, Ballarat, Australia; 2 Institute for Health and Sport, Victoria University, Footscray, Australia

**Keywords:** Sport participation, Leisure-time physical activity, Settings

## Abstract

**Background:**

Leisure-time physical activity and sport participation trends are often reported, both in aggregate and by specific activity. Recently there has been a rise in overall leisure-time physical activity, but little change in the prevalence of organised sport. It is important that the development of sport policy, infrastructure and strategic developments meet the changing landscape of participation. However, there has been relatively little research into the settings in which people participate. The aim of this study is to investigate the settings of participation of children and adults in 12 major Australian sports.

**Methods:**

This study utilised data about participation in sport and recreational physical activity collected in the AusPlay survey from a representative sample of adults and children in the Australian state of Victoria. For each type of physical activity, the settings of participation are identified. Respondents can report participation in a particular activity in more than one setting. Therefore we use the term “instance of participation” to refer to a person playing a particular sport in a particular setting. Participation and settings across 12 major sports were investigated for children and adults.

**Results:**

For children, the most popular sport was swimming with a weighted estimate of 323,565 (30.3%) instances of participation in the Victorian population, followed by Australian football (*n* = 180,459; 16.9%), and basketball (*n* = 137,169; 12.9%). For adults the most popular sports were swimming (*n* = 703,950; 30.9%) followed by golf (*n* = 274,729; 12.1%), and tennis (*n* = 260,814; 11.4%). There were considerable differences between the profiles of settings of participation for the 12 sports. Across the 12 sports, the majority of participation by children took place within a sports club or association setting, representing 63% of all instances of sport participation. For adults, sports clubs and associations was also the most popular setting, but it represented only 37% of instances of participation.

**Conclusions:**

Traditionally, community clubs and inter-club competitions provided the main setting for sport participation, but this is no longer the case, particularly for adults. If the community sport sector is to continue to flourish, it must consider new strategies and participation options more attractive to other segments of its potential market.

## Background

Internationally, sport policy often has two participation focuses, elite performance, and community level participation [1, 2]. The national Australian sport policy has recently redefined the definition of sport to be more inclusive of a wider range of physical activities. Until recently, in Australia sport was defined as “*A human activity capable of achieving a result requiring physical exertion and/or physical skill which, by its nature and organisation, is competitive and is generally accepted as being a sport”* [3]*.* However, recently Sport Australia developed a new plan, Sport 2030 [1]. In this policy, “sport” was redefined as an umbrella term for recreational physical activity, which is more consistent with established use of the term internationally. The new definition of sport is broader, including both organised competitive sports as previously, but also encompassing a broad range of other recreational physical activities, such as walking, riding, swimming, “ninja’ style obstacle courses and stand-up paddle boarding [1].

This definition is more in line with many other countries which have long since adopted a broad definition of sport which includes organised and non-organised sport, as well as other leisure-time physical activity. For example, Sport England’s strategy is ‘towards an active nation’ and includes people’s engagement in sport and physical activity [4]. In New Zealand, there is a separate strategy for community sport and their definition includes sport and recreation [2]. More specifically, in New Zealand community sport includes play, active and outdoor recreation and competitive sport within sports clubs and events. This definition specifically excludes passive recreation such as gardening, and also excludes elite (international) competition [2].

Even though the definition of sport varies internationally, the key policy aim is consistently to increase participation [1, 2, 4]. Specifically the Australian plan is to have “more people of all ages engaged in sport and physical activity throughout every stage of their life” (p4) [1]. With a policy focus on increasing participation, research has often investigated the trends in sport participation [5–7]. There is evidence from a longitudinal study of adults in Germany that participation in sport, defined as participating at least weekly over the past 6 months, has increased over past decades [5]. In the Australian state of Victoria, participation in five major sports reportedly increased by over 50,000 participants over a 3-year period, from 414,167 (prevalence 7.5%) in 2010 to 465,403 (prevalence 8.3%) in 2012 [6]. Furthermore, in Flanders, Belgium participation in sport for those aged 13–18 years increased from 45% in 1989 to 54% in 2009 [8]. However, in France participation in sport by those of high school age reportedly decreased from 79% in 2001 to 66% in 2015 [7].

More specifically related to particular types of activities, a recent Australian study of trends over 10 years (2001–2010) reported that whilst the rate of participation in fitness activities (such as gymnasium workout, aerobics and treadmill), increased from 13 to 24%, participation in holistic movement practices (such as yoga and Pilates) remained stable [9]. Another study utilising the same Australian national survey data, reported that for muscle-strengthening activities, the rate of sufficient muscle strengthening activity significantly increased over 10 years from 6.4 to 12% [10]. A systematic review and meta-analysis of global participation in sport and physical activity, whilst not reporting trends over time, did point out that types of participation differ across age and regions [11]. For example, walking was the most popular physical activity for adults, whereas for adolescents it was soccer, running, walking and athletics and for children running and soccer [11].

We also know that the types of activities people participate in have changed considerably over time. For example, a 10-year Australian study of leisure-time physical activity among those aged 15 years and over reported that whilst participation rates in any leisure-time physical activity increased significantly over the decade, there was no increase in organised and/or club-based participation [12]. Similarly, a 30-year observational study in Germany reported that participation in competitive sport has decreased while participation in informal sport has increased [5]. There are also differences in the types of sport and physical activity participation across the lifespan, and between genders. For example males are more likely to participate in organised club-based sport than females [5, 13], and people are more likely to participate in organised club-based sport as children rather than as adults [14].

Australian sport policy has recognised that the sporting landscape has changed and is continually evolving. In accordance with this, the sport sector needs to develop and adapt to meet the present and future challenges, and offer sport that meets the needs of the public [1]. For example, there have been in recent years developments of social-recreational based sports programs and products for the adolescent and adult market, which are quite distinct from the traditional club-based competitive sport model. For example, VicHealth in Australia has recently been funding sports to develop innovative products that are more flexible, social and less structured [15]. On the consumer side, the motivations for participation in sport have been shown to differ between public and private run sports facilities [16].

Therefore, the settings for participation are now broader and more diverse than the traditional local community sports club, but as yet little research has been conducted into the extent of these changes or the different impacts on different sports.

It is also important to consider participation patterns across the lifespan. National sport policy recognises that people’s participation across the lifespan differs with regard to the type of activity, frequency and duration of participation [17]. For example there is a marked tendency for those transitioning from childhood to adolescence and young adulthood to move from participation in organised club-based sport to participation in non-club settings [18, 19].

Participation in sport is changing in Australia. Trends in leisure-time physical activity and sport participation are often reported, together with participation rates per activity. However, we do not have clear knowledge of the settings in which people participate. Individual sports generally capture participation data from their organised, competitive club-based players but not social or recreational players, who may play their sport but not within the traditional club structure [20]. It is important that the development of sport policy, infrastructure and strategies meets the changing landscape of participation and that sport organisations understand the total picture of participation in sport across different settings, and the implications for their operations and their provision of facilities and programs.

The aim of this study is to investigate the settings of participation of children and adults in major Australian sports.

## Methods

We investigated the settings of participation in 12 major Australian sports. These sports cover the majority of organised, club-based sport participation in Australia, and the research team are conducting a range of research projects focused on these 12 sports (see www.sportandrecreationspatial.com.au).

The former national Exercise, Recreation and Sport Survey, commissioned by the Australian Sports Commission, now Sport Australia, was discontinued in 2010. After a gap of almost 5 years, and following the Australian Bureau of Statistics’ decision in June 2014 to cease funding for all sport and recreation data collection, the ASC-commissioned AusPlay survey (AusPlay) was commenced in the last quarter of 2015.

A detailed description of the AusPlay methodology as used in 2016–2017 can be found elsewhere [21]. Briefly, the target population for AusPlay is all Australian residents. Randomly selected Australian residents aged 15 years and over are interviewed directly in a computer aided telephone interview (CATI). In AusPlay parlance, the term “adult” is applied to this sample. While the application of this term to persons as young as 15 years may be questionable, it provides a simpler alternative to repetitive use of a phrase such as “persons aged 15 years or more”, and it distinguishes the main survey sample from the secondary sample of “children” (aged 0–14 years). A more restricted set of data about children (with questions on topics such as motivation and costs of participation being omitted) is collected from adult respondents who are parents or guardians of at least one child in their household, with these respondents also providing data about one randomly selected child.

The annual target sample size for the AusPlay survey is 20,000 adults (aged 15 years and over) spread equally across the year, with 5000 adult interviews being conducted each quarter. The AusPlay sample is stratified, with the overall target of 5000 adult interviews being split into target sample sizes for each of 13 geographic strata based on States and Territories with further splits into the Greater Capital City Statistical Areas and the Rest of the State in the case of New South Wales, Victoria, Queensland, South Australia and West Australia. For selecting the adult sample, an overlapping dual-frame approach was used, with a stratified sampling frame for fixed landlines and a separate frame for mobile phones, with random digit dialling within each frame, and random selection of respondents within each contacted household in the fixed landline sample.

The sample of respondents contacted in this way within each stratum (region) is unlikely to be representative of the population of the region in important ways, such as age and gender profiles. In order to ensure that the estimated counts and rates for the whole population based on the sample data are representative of the whole population, the data from each AusPlay respondent is assigned a weight. The weights were based on the estimated resident population [22] in each of the 156 “cells” of a 3-factor classification: geographical region (13) × gender (2) × age (6). In principle, responses from respondents in cells which are under-represented in the AusPlay sample (relative to the population) are up-weighted (multiplied by a weight > 1) and responses from cells which are over-represented in the AusPlay sample are down-weighted (multiplied by a weight < 1). In practice, the weights must also include adjustments for household size and for other complexities relating to the combining of two sampling strategies. Finally, weights are rescaled so that the sum of the weights for each quarterly sample is equal to the population of Australia (aged 15 years and over). The weights also sum to the population counts in each cell of the cross-classification. Consequently, weighted sample estimates of the numbers of persons with a particular characteristic (such as playing a particular sport) are direct estimates for the population or relevant sub-population. To maintain these properties, when data from more than one quarter are aggregated, the weights are divided by the number of quarters. Weights for child data are determined by a similar process, but included further adjustments for the number of children in the household. The weighting methodology is described in more detail in the AusPlay methodology report [21].

The sampling uncertainty in estimated counts and rates (e.g. the number and percentage of women in Victoria aged 25–44 who play netball in non-club settings) can be expressed in terms of four measures: standard error, relative standard error, margin of error, or relative margin of error. These measures depend on the relevant sample size, and hence on the size of the estimate; the larger the estimated count, the larger the error but the smaller the relative error. These measures are tabulated and explained in detail in the AusPlay methodology report [21].

The flow of the AusPlay interview and the questions asked are shown in two other AusPlay publications [23, 24]. After initial demographic questions, respondents were asked whether they had participated in any physical activity, for sport, exercise or recreation during the 12 months prior to the interview. Those who had done so were invited to nominate up to 10 types of physical activity (e.g. basketball, tennis, aerobics, walking). The scope included both sports as defined in Australia at the time (see Introduction above) [1] and other forms of recreational physical activity. For each physical activity type nominated, participants were then asked a number of further questions about the frequency, duration and the settings in which the activity occurred. In accordance with the aims of the present study, our focus is on the settings reported for each of the 12 sports included in the study [24] p4.

For each type of physical activity, respondents were first asked *“In the last 12 months, did you do any of this through an organisation – like a club or a gym; or at a venue – like a pool or an oval?”* Respondents who answered “Yes – all” or “Yes – some” were asked to indicate what types organisations or venues from a list of 12 types of setting. In the present study, to keep tabulations within manageable proportions, these 12 types were collapsed into five broader settings, as shown in Table 1, together with the sixth setting “Not organised”. Physical activity within school hours and informal recreational physical activity not involving an organisation or venue were excluded from the scope of data collection for children aged under 15 years [23] p17. Consequently, the “Not organised” setting does not apply to children, and the “Work, education” setting for children includes only activities organised by schools but undertaken outside school hours.

**Table 1:** Setting categories

Collapsed category	Original categories
Sports club or association	• Sports club or association
Gyms, centres etc.	• Gym/Fitness club/sports/leisure centre • Private studio (e.g. dance, yoga, pilates, martial arts) • Individual personal trainer or coach
Community, rec clubs etc.	• Recreation club or association (e.g. social club, senior citizens’ club, abseiling association) • Public space (including park, oval, beach) • Events (e.g. fun run or Parkrun) • Community-run programs
Work, education	• Work • Educational institution (e.g. school or university)
Other, don’t know	• Other (record answer) • Don’t know
Not organised	

### Statistical analysis

AusPlay data for the state of Victoria collected in the four quarters of 2017 were analysed for the present study. For each of the 12 included sports, we summed the weighted survey responses pertaining to each of the five (children) or six (adults) broad settings listed in Table 1. Because the weights are designed to scale estimates up to population level, the estimated counts tabulated below and the percentages and prevalences based on them are estimates for the Victorian population in the two age groups.

Counting the extent of participation in each setting for each sport is complicated by the fact some respondents participated in a particular sport in more than one setting. In this study, we have used the term “an instance of participation” to refer to a person playing a particular sport in a particular setting. An “instance” is not an episode/occasion/session/match; frequency of participation is not the focus of this study. Nor does an instance necessarily represent participation at a single location. For example, a respondent aged 17 who played basketball during the previous 12 months in two settings, such as a community club team and at school, contributes two instances of basketball participation, one for each setting, regardless of the number of occasions or locations involved in each setting.

## Results

The AusPlay sample sizes for the state of Victoria for the four quarters of calendar year 2017 were 4210 adults (aged 15+) and 550 children (aged < 15). Of those, 1653 adults (aged 15+) and 488 children (aged < 15) played one or more of the 12 sports included in the study. Table 2 shows unweighted counts by gender and age.

**Table 2:** AusPlay survey 2017: sample characteristics for Victoria^a^

	AusPlay sample			Study sample^b^		
Age (years)	Male	Female	Total	Male	Female	Total
4	20	21	41	18	16	34
5–9	127	103	230	122	92	214
10–14	141	138	279	130	110	240
Children (4–14)	288	262	550	270	218	488
15–19	159	110	269	118	63	181
20–24	174	159	333	107	68	175
25–29	178	130	308	110	34	144
30–34	172	114	286	91	43	134
35–39	166	161	327	73	52	125
40–44	160	178	338	82	61	143
45–49	157	185	342	69	53	122
50–54	166	177	343	65	50	115
55–59	163	170	333	66	49	115
60–64	153	204	357	61	58	119
65–69	158	219	377	62	61	123
70–74	114	172	286	42	29	71
75–79	89	89	178	31	18	49
80+	56	77	133	18	19	37
Adult (15+)	2065	2145	4210	995	658	1653

^a^Unweighted frequencies

^b^AusPlay survey respondents who reported playing any of the 12 sports included in the study in the 12 months prior to the survey interview

Tables 3 and 4 provide summaries of the estimated counts and percentages of participation instances across the settings, for children and adults respectively. As explained under Statistical Analysis above, the total number of instances of participation in each sport (the row totals in Tables 3 and 4) is greater than the number of participants in the sport, although the excess is small (< 1%) because the great majority of respondents participated in a particular sport in only one setting. Consequently the percentages of instances in each row of Tables 3 and 4 are slightly conservative estimates of the percentage of participants in each sport who play in each setting. However, the shape of the profile across the settings is not affected substantially. When the data for the 12 sports are aggregated (the bottom row of each table) the multiple counting effect is greater because many respondents participated in more than one sport.

**Table 3:** Estimated instances of participation^a^ in 12 sports in 2016–2017 by Victorians aged < 15 years: by sport and setting

	Setting												Aggregate (5 settings)^d^	
	Sports clubs & associations		Gyms, centres etc.		Community, rec clubs etc.		Work, education^b^		Other, don’t know		Not organised^c^			
Sport	Count	%	Count	%	Count	%	Count	%	Count	%	Count	%	Count	Rank
Australian Football	161,101	89			10,126	6	6809	4	2423	1			180,459	2
Basketball	120,104	88			5804	4	9932	7	1329	1			137,169	3
Bowls														
Cricket	62,670	88			903	1	5324	7	2544	4			71,441	8
Golf	12,301	80	1774	12	1225	8							15,300	10
Gymnastics	19,983	28	24,110	34	15,726	22	3449	5	8554	12			71,822	7
Hockey	15,827	85					2750	15					18,577	9
Netball	74,787	92	376	< 1			5810	7					80,973	5
Sailing	1570	100											1570	11
Soccer	67,103	85			8327	10	2013	3	1913	2			79,356	6
Swimming	63,907	20	158,717	49	35,695	11	24,382	8	40,864	13			323,565	1
Tennis	69,452	81	9294	11	740	1	937	1	5544	6			85,967	4
Aggregate (12 sports)^e^	668,805	63	194,271	18	78,546	7	61,406	6	63,171	6			1,066,199	

^a^An instance of participation is participation by a person in a particular sport in a particular setting

^b^For children (< 15) the “Work, education” setting includes only activities organised by schools but undertaken outside school hours

^c^The “Not organised” category applies only to adults (15+ yrs)

^d^The row aggregates are calculated for each sport by summing the counts for the six settings. Because a small proportion of respondents reported playing a particular sport in more than one setting, the aggregate figures are slightly (< 1%) higher than the total number of participants for each sport

^e^The aggregates for each setting are calculated by summing the counts for the 12 sports. Because some respondents reported playing more than one sport in a particular setting, the aggregate figures are higher than the total number of participants for each setting and for all settings combined

**Table 4:** Estimated instances of participation^a^ in 12 sports during 2016–2017 by Victorians aged 15+ years: by sport and setting

	Setting																		Aggregate (6 settings)^c^	
	Sports clubs & associations			Gyms, centres etc.			Community, rec clubs etc.			Work, education			Other, don’t know			Not organised^b^				
Sport	Count	%	%^b^	Count	%	%^b^	Count	%	%^b^	Count	%	%^b^	Count	%	%^b^	Count	%		Count	Rank
Australian Football	101,232	54	73	21,795	12	16	7543	4	5	7075	4	5	283	< 1	< 1	50,777	27		188,705	6
Basketball	83,258	37	55	30,596	14	20	14,734	7	10	14,836	7	10	9053	4	6	73,429	33		225,906	4
Bowls	28,144	52	53	5695	11	11	15,581	29	29				3729	7	7	834	2		53,983	9
Cricket	75,029	56	71	10,275	8	10	7364	6	7	10,411	8	10	1954	1	2	27,910	21		132,943	8
Golf	167,474	61	70	28,524	10	12	28,141	10	12				13,956	5	6	36,634	13		274,729	2
Gymnastics	2827	18	22	6577	42	50	1049	7	8	2647	17	20				2594	17		15,694	12
Hockey	32,225	64	67	11,522	23	24				4475	9	9				1879	4		50,101	10
Netball	86,294	60	65	26,434	18	20	10,455	7	8	4524	3	3	5413	4	4	11,404	8		144,524	7
Sailing	12,399	65	77	1853	10	12	1775	9	11							3081	16		19,108	11
Soccer	79,896	38	57	30,821	15	22	11,499	6	8	13,495	6	10	5638	3	4	66,633	32		207,982	5
Swimming	61,192	9	13	242,872	35	53	90,009	13	20	24,388	3	5	38,002	5	8	247,487	35		703,950	1
Tennis	118,677	46	65	28,381	11	15	18,836	7	10	5914	2	3	11,460	4	6	77,546	30		260,814	3
Aggregate (12 sports)	848,647	37	48	445,345	20	29	206,986	9	13	87,765	4	4	89,488	4	6	600,208	26		2,278,439	

^a^An instance of participation is participation by a person in a particular sport in a particular setting

^b^The “Not organised” category applies only to adults (15+ yrs). For comparison with Table 3, the second column of percentages for each organised setting is calculated with reference to the total count for the five organised settings, with the “Not organised” setting excluded

^c^The row aggregates are calculated for each sport by summing the counts for the six settings. Because a small proportion of respondents reported playing a particular sport in more than one setting, the aggregate figures are slightly (< 1%) higher than the total number of participants for each sport

The aggregates for each setting are calculated by summing the counts for the 12 sports. Because some respondents reported playing more than one sport in a particular setting, the aggregate figures are higher than the total number of participants for each setting and for all settings combined

For children, there were an estimated 1,066,199 instances of sport participation in Victoria across the 12 sports and the five settings of organised participation (Table 3). There were twice as many instances of participation for adults, across the six different settings (*n* = 2,278,439) (Table 4). For children the most popular sport was swimming with 323,565 instances of participation followed by Australian football (*n* = 180,459) and then basketball (*n* = 137,169) (Table 3). For adults the most popular sports were swimming (*n* = 703,950) followed by golf (*n* = 274,729) and then tennis (*n* = 260,814) (Table 4).

For children the great majority of participation took place within a sports club or association setting. This represented 63% of all instances of sport participation (Table 3). The next most popular setting for children’s organised sport participation was a gymnasium, representing 18% of instances of participation. For adults, sports clubs and associations was also the most popular setting for participation, representing 37% of instances of participation (Table 4). A further 26% of participation was not organised and 20% was within a gymnasium.

There were considerable differences between the 12 sports in the profiles of settings of participation. For children, 10 sports (Australian Football, basketball, cricket, golf, hockey, netball, soccer and tennis) had 80% of more of their participation instances within a sports club or association (Table 3). For gymnastics and swimming, these types of setting represented only 28 and 20% respectively. The most common setting of participation for gymnastics and swimming was a gymnasium, representing 34 and 49% respectively. Three sports – gymnastics, swimming and tennis – had participation across all five settings. Conversely, within community and recreational clubs the sport most represented was gymnastics, followed by swimming and soccer. Within the work and education setting, the sport most represented was hockey followed by swimming.

For adults, seven sports (Australian Football, bowls, cricket, golf, hockey, netball, and sailing) had more than 50% of their participation within a sports club or association (Table 4). Only 9% of swimming, 37% of basketball and 38% of soccer participation instances for adults was within a sports club or association. Overall, the most popular setting was sports club or association (37%), followed by not-organised (26%) and gymnasium (20%). Eight sports (basketball, cricket, netball, soccer, swimming and tennis) had participation across all six settings. All 12 sports had participation within the gymnasium settings. Conversely, within gymnasium settings the sports most represented were gymnastics and swimming. Within community and recreational clubs the sports most represented were bowls and swimming. Within work and education settings the sports most represented were gymnastics and hockey. Within the not-organised setting the sports most represented were swimming, basketball, soccer and tennis.

For purposes of comparison between the participation settings of children and adults, Table 4 also provides percentages of organised sport settings for adults i.e. with non-organised settings excluded. Children were more likely to participate in sport within a sports club or association (63%) than adults (48%). Adults were more likely then children to participate in sport through gymnasiums (29%) and community recreation clubs (13%) than children (18 and 7%). Children were slightly more likely to participate in sport through education/work (6%) than adults (4%).

## Discussion

There is much research investigating the types of leisure-time physical activities that people participate in, as well as the frequency and duration of participation [13, 25, 26]. This health promotion-based research focuses on trying to get the population active and at recommended levels for health [27]. However, it is also important to understand the settings of participation, that is, where people chose to be active. This is important for both health promotion and sports management strategies. This study uniquely investigated participation settings across 12 major Australian sports and the profile of settings of organised sport participation for children and adults. It clearly demonstrates that there are great differences in the profiles of participation settings between different sports and between children and adults. The range of available settings for sport participation has been broadening for some time, but while specific aspects of settings have been addressed in passing in some research studies [16, 18, 19], this has not been the primary focus of any previous research.

Overall, for children the most popular of the 12 sports were swimming, Australian Football and basketball, and for adults swimming, golf and tennis. Given the Australian culture and considering that the measure of participation was at least once in the past 12 months, it is not surprising that swimming was the highest participation sport. Furthermore, swimming for adults was the highest not-organised activity. Swimming is a popular leisure-activity that does not need to be organised, nor does it require formal clubs and associations, as demonstrated in this study. Of all leisure-time physical activities, participated at least once in the past 12 months, participation in swimming is the most popular activity for children, with 30% of Australian children participating in swimming activities [28].

The great majority of the children’s participation in sport was through a local sports club or association, followed by gymnasium, and with only a few instances of participation in the other settings, with the exception of gymnastics and swimming. In comparison, adults were less likely to participate in sport through a sports club or association than children, and were more likely to participate across many different settings. Participation of adults was higher within the gymnasium and the community and recreation club settings than for children. For some sports organised participation occurred across multiple settings, whilst in other cases one or two settings predominated.

We know that the way people are active throughout their life changes considerably [13, 29]. Participation in club-based sport is popular, however mainly for children and young adolescents [14]. The great majority of people drop out of organised, competitive club-based sport at some stage throughout their life. There is much literature on drop-out of participation in sport [30–33], and also evidence that participation in sport is associated with access to local facilities [34–36]. This study is unique in that it demonstrates that many adults still play the traditional club-based sports, but not only within traditional club-based settings. Given this, it is important for future research to investigate why adults choose to participate in sport through settings other than sports clubs. Perhaps it is the traditional competitive model of sport delivered through club settings that is not conducive to adult participation. Or is it that the sport-club offerings are really only for children and youth and young adults, with comparable age, skills, competition and fitness levels? There is recent evidence that the motivations of sport participants for adults differed according to the setting of participation [37]. For example, those adults who participated in sport in sports clubs were motivated by extrinsic goals related to image, as well as intrinsic goals related to skill development and social affiliation, whereas adults participating in sport through informal setting such as public spaces were more motivated by health-related goals [37].

Perhaps the development of more flexible and less competitive options may be more suitable for many adults. There is also the fact that sports clubs are generally run by volunteers and are quite structured in their times of training and play, and perhaps other settings provide more flexibility regarding times and more professional services that adults prefer. Furthermore, many commercial gymnasiums and other commercial settings do not allow children to participate.

There has been a rise in participation in leisure-time physical activity in general, however this has not been matched by any increased in organised participation within a club setting [12]. In line with this evidence of participation changes, Sport Australia have acknowledged that the consumer needs for sport participation are changing and that the sport sector needs to adapt to the changing needs [38, 39]. Further, there has been a rise in strategic prioritising, including funding and program development, of the social and informal or less structured versions of sport and active recreation [40].

National and State Sporting Associations are the governing bodies of their individual sports in Australia. These organisations generally only govern registered club-based participants, although there is in some cases an increasing understanding of and focus on alternative social recreation program options. Given that these organisations do not govern participation in their sport in other settings, including commercial providers, which have grown in recent years, it is in their interests, and also imperative for responsible government departments and public sector organisations such as Sport Australia, State Governments and VicHealth, to turn more of their attention to the market segment, particularly amongst adults, who are turning to avenues other than traditional community club sports competitions to provide their sporting and recreational experiences.

From a national perspective, there is a need for sport policy to consider private providers as well as traditional sporting clubs, with umbrella strategies and polices to cater for the wider population rather than just those, mainly children and youth, who participate in the traditional sports club. From the perspective of the sports organisations, it is important to understand and respond to the trend towards participation in their sport across a spectrum of different settings, and not just focus on those registered to play in club-based competitions and programs.

A strength of this study is that, being based on a large state-wide sample from a very large national survey, the sampling errors in the estimated percentages in the settings profiles are small, and so from that perspective the results are very reliable. Furthermore, whereas its predecessor in Australia, the Exercise, Recreation and Sport Survey was limited to persons aged 15 years or more, in the AusPlay survey data are also collected about children younger than 15 years. This is important because, as the results demonstrate, the profiles of participation settings of the two age groups are very different.

A potential limitation to this study is that the measure of participation used is very basic, “at least once in the last 12 months”, which does not distinguish between a single session and weekly or daily participation. The reason for this is that, because the focus of this paper is on settings of participation, we were constrained by the form in which data about settings is collected in the AusPlay survey. While data about frequency of participation are collected for each sport played by a respondent, these data are not collected separately for each setting in which the sport is played. In principle, to derive a more sensitive frequency-based measure of participation for those respondents who played a particular sport in multiple settings, frequencies for each setting would have to be imputed, which would require ad hoc decisions on a case-by-case basis, which would inject further random errors into the estimates over and above sampling error. We opted not to do this in the present study. It is possible that frequency of participation in a particular sport may differ for different settings, in which case the resulting profiles may have been different, but this is not necessarily the case.

Finally, the possibility of non-sampling errors, in particular response bias, is a perennial issue for all survey-based research. A recent study compared 2010 ERASS estimates of club-based participation in four sports in Victoria with corresponding 2010 counts of registered participants. The 2010 ERASS survey data were found to massively over-estimate the prevalences of club-based participation in four major sports. It was concluded that this was because of response bias, whereby participants were more likely to agree to take part in the survey than were non-participants [20]. Given the similarity of the survey methodologies employed in ERASS and AusPlay, it is reasonable to expect that similar response bias would be present in AusPlay estimates of prevalence. However, the focus of this study was not prevalence of participation, but rather settings of participation. Even in the presence of pronounced response bias, conclusions about cross-sectional comparisons and correlations remain valid to the extent that the response bias is consistent across the scope of the data analysed, in this case the different participation settings.

## Conclusion

In conclusion, most children and many adults play sport, but the settings in which they participate or play are quite different across age and across sports. Firstly, it is recommended that the reasons why adults choose to play sport through settings other than traditional clubs is further investigated. Secondly it is recommended that sport considers the market segment particularly amongst adults, who participate in sport through settings other than community sports clubs, and consider new strategies and participation options more attractive to this segment of its potential market.

## Data Availability

The data were provided by Sport Australia and access to the data would need to be sought from Sport Australia.
